# Energy and vulnerability: Exploring the energy poverty-risky sexual behavior nexus among young women in Ghana

**DOI:** 10.1016/j.ssmph.2023.101597

**Published:** 2024-01-02

**Authors:** Michael Adu Okyere, Aaron Kobina Christian, Felix Essel-Gaisey, Fawzia Muhammed Zuka

**Affiliations:** a Clean Air Task Force, Boston, MA, 02109, United States; b Regional Institute for Population Studies, University of Ghana, Legon- Accra, P. O. Box LG 96, Accra, Ghana; c School of Economics, Henan University, Kaifeng, 475004, Henan, China; d Center for Coastal Management, Africa Centre of Excellence in Coastal Resilience, Department of Fisheries and Aquatic Sciences, University of Cape Coast, Ghana

**Keywords:** Multidimensional energy poverty, Risky sexual behavior, Lewbel 2SLS, Developing country, Demographic and health survey, Resource deprivation

## Abstract

While recent policy agendas have extensively addressed both energy poverty and risky sexual behavior separately, little research has explored their intersection. This pioneering study investigates the relationship between energy deprivation and risky sexual behavior in female youth. Utilizing data from the Demographic and Health Survey in Ghana and employing robust methodologies, including the Lewbel two-stage regression and alternative energy deprivation thresholds, our findings reveal an 8.2–8.7% increase in the likelihood of female youth engaging in risky sexual activities for each percentage increase in energy poverty. Notably, these estimates hold consistent across different wealth terciles and location subgroups. Furthermore, we identify the number of lifetime partners as a potential mediator through which energy poverty influences risky sexual behavior. In conclusion, our study emphasizes the urgent need for policymakers to prioritize the adoption of affordable energy services, particularly among impoverished and rural households. Such efforts have the potential to mitigate the adverse effects of energy poverty on risky sexual behavior. We also advocate for the integration of energy poverty eradication strategies within reproductive and sexual health programs to foster comprehensive development and well-being.

## Highlights

• Young women who are energy poor are more likely to engaged in risky sex.
• Energy poverty consistently influences young women's sexual behavior across wealth and location subgroups.
• The nexus between energy poverty and risky sex is mediated by the number of partners.

## 1 Introduction

The energy sector occupies a pivotal role within the global economy, meriting its inclusion in the 2030 Agenda for Sustainable Development, with a specific focus on Sustainable Development Goal 7. From a policy standpoint, the progress within this sector is frequently intertwined with economic growth, energy security, and addressing climate change concerns. Regrettably, the concept of energy poverty, characterized by the inability to access essential energy services, energy inputs, outcomes of energy use, and the quality of energy (González-Eguino, 2015; Culver, 2017), has been demonstrated to yield substantial and far-reaching consequences. The different aspects of energy services often considered include access to electricity, non-biofuel for cooking, energy for entertainment and communication. These consequences extend across areas such as education, health, and subjective well-being, as underscored in prior research (Zhang et al., 2021; Churchill et al., 2020; Lin & Okyere, 2020; Thomson et al., 2017; Biermann, 2016; Lin & Okyere, 2021). While research examining the consequence of energy poverty has gained momentum, a notable gap in the existing scholarly discourse revolves around our understanding of the potential impact of energy poverty on individual behavioral patterns, for example risky sexual behavior.

Risky sexual behavior stands as a prominent risk factor in the global burden of disease (Chan, 2021). Risky sexual behavior does not only have health implications but also has a wide range of social consequences (Adhikari, 2009). Contemporary public health policies emphasize the promotion of safe sexual practices and education over the prohibition of sexual activities, giving rise to growing concerns about the alarming rates of unintended pregnancies and sexually transmitted infections (STIs), notably in sub-Saharan Africa (Herman et al., 2017). This concerning trend is particularly pronounced among adolescents and marginalized young individuals, prompting inquiries into the root causes of risky sexual behavior (Ngoc Do et al., 2020). While previous research has indicated that resource scarcity plays a significant role, empirical studies addressing specific forms of deprivation remain limited in the literature. In light of this, our study conducts an empirical investigation into the impact of a particular form of deprivation—energy poverty—on risky sexual behavior among young females and explores the key pathways through which it exerts influence.

This study specifically focuses on female youth as they suffer most from the implications of gender-based inequalities such as resource deprivation as asserted by the theory of Gender and Power (Connel, 1987; Wingood & DiClemente, 2000). Females are often considered to be highly likely to contract STIs and suffer from sexual relationship inequalities, such as the fear of being physically abused in condom use negotiations (Raiford et al., 2009; Spiegel & Futterman, 2009; Torian et al., 2011; Wingood & DiClemente, 2000). Compared to their male counterparts, female youth have lower condom use and risk perception of STIs and a higher rate of indulging in risky sexual practices (Christian et al., 2021; DiClemente et al., 1996). Furthermore, female youth with high levels of psychological distress are prone to STIs, engage in sexual acts while high on drugs, concurrently have male and female partners, and are very fearful when communicating with their partners (Seth et al., 2011). Therefore, a study that unearths the extent to which a specific deprivation such as energy poverty affects the sexual behavior of this group may significantly impact the formulation of policies that seek to improve their welfare.

This study makes several contributions to the literature. The first strand adds to the literature on risky activities and resource deprivation. These studies usually posit that being deprived increases one's chances of getting involved in dangerous activities such as crime, unsafe sex, and violence (Christian et al., 2021; McIlwaine & Moser, 2003; Noar et al., 2012). However, they usually fail to account for the specificity of deprivation. As energy poverty forms a distinguishable aspect of resource deprivation (Moore, 2012), we build on this literary feature by concentrating mainly on the effect of energy deprivation on risky sexual behavior. Supported by Crutchfield and Wadsworth (2003), an analysis that specifically understudies specific resource deficiencies helps in the granular analysis and aids policy formulation and implementation. Furthermore, we add to the scope of literature on the effects of energy deprivation (Amin et al., 2020; Churchill et al., 2020; Lin & Okyere, 2020, Lin & Okyere, 2021). These studies have examined the effect of energy poverty on economic development, subjective well-being, and health. The context on health outcomes has primarily focused on the effects of energy poverty on mental influences (Churchill et al., 2020; Lin & Okyere, 2020; Thomson et al., 2017). On the contrary, we contribute to these studies by explicitly focusing on risky sexual activities – a dimension of physical health disorder. Thirdly, this study leverages one of the most comprehensive microdata collections pertaining to households in Ghana to probe the mechanisms underlying the impact of energy deprivation on risky sexual behavior. Access to microdata encompassing both energy utilization and sexual behaviors in developing economies is often a formidable challenge, imposing constraints on related research endeavors. The Demographic and Health Survey (DHS) data used in this paper is the largest publicly available dataset on household energy services and health and allows us to control for rich household characteristics to reduce estimation bias. More importantly, this dataset contains several individual lifestyle variables on which we can delve into the mechanisms or channels through which energy poverty affects risky sexual activities among females.

The rest of the study is organized as follows: the next section discusses the conceptual framework of how energy poverty affects risky sexual behaviors. Section 3 explains why the study is relevant in Ghana; Section 4 describes the data, variables, and estimation strategy; Section 5 presents the results and discussion, and Section 6 gives the conclusion and implications.

## 2 Conceptual framework and hypotheses

This section provides a conceptual analysis and hypothesis of how the lack of energy access can affect risky sexual behaviors among female youth.

The Broken Window theory, originally used in examining community disorder and crime (Wilson & Kelling, 1982), can help explain the relationship between energy poverty and risky sexual activities. The theory posits that people's actions may be inspired via messages their physical surroundings signal. By this theory, a chaotic and disordered surrounding characterized by faulty street lights, frequent blackouts, among others, may signal occupants that prohibited behaviors such as prostitution, illicit drug use, and vandalism are permissible and may be normative over time. Therefore, in environments where people live in less insulated structures without grid connections, faulty or unavailable street lights, risky sexual conduct is deemed normal (Jessel et al., 2019). However, some studies have critiqued this theory as having some conceptual and methodological issues. For example, Sampson and Raudenbush (2001) contend that this theory assumes disorder as a vital and standard phenomenon and argues how the idea has neglected cultural stereotypes and context. That notwithstanding, studies that have applied this theory have observed higher rates of sexually transmitted infections among residents in these risky neighborhoods (Cohen et al., 2000).

Furthermore, the lack of proper sexual information sources facilitated via end-use appliances is another perspective on how energy poverty can affect risky sexual behavior (Awusabo-Asare & Annim, 2008; Latifnejad Roudsari et al., 2012; Mirzaii Nagmabadi et al., 2014). Thus, deprived people usually struggle to use their resources to obtain credible information to take protective measures, such as seeking treatments for sexual infections or using a condom (Lopman et al., 2007). Consequently, a deprivation in end-use appliances such as television or radio deprives people of information on the dangers of getting involved in risky sexual activities, thereby increasing their chances of opting for riskier sex. We, therefore, hypothesize that energy deprivation increases the likelihood of females engaging in risky sexual activity.

Hypothesis A Energy poverty positively influences risky sexual behaviors among females.

Qualitative and anecdotal studies suggest that deprivation of resources increases the chances of females engaging in risky sexual acts through multiple partners (Khan et al., 2010; Silas, 2013). Thus, when women are deprived of energy services, they are likely to have numerous sexual partners (Mill & Anarfi, 2002; Weiser et al., 2007). For example, with energy access deemed as a means of enhancing social status (Day et al., 2016; Lin & Okyere, 2021; Middlemiss et al., 2019), people, especially females, are likely to date multiple men to enable them to afford energy services to reinforce their positions in society (Khan et al., 2010). Similarly, with energy poverty noted to result in anxiety or depression (Churchill et al., 2020; Lin & Okyere, 2021; Zhang et al., 2021), females who are sometimes overwhelmed with the stress presented by their inability to access energy services resort to having multiple partners as a maladaptive strategy to cope with or regulate the effect (Briere, 2004). Therefore, when females are energy-deprived, their numerous partners serve as a resource that can be drawn upon for material or economic advantage. Consequently, having many partners increases one's likelihood of engaging in riskier sexual acts. This is because safe sex may be expensive and warrant welfare losses if energy resources provided by the numerous partners of the female's compensation for the risk of having unprotected sex. We therefore hypothesize that, the total number of lifetime partners for a female mediates the relationship between energy access and risky sexual activity.

Hypothesis B Total lifetime partners mediate the positive relationship between energy poverty and risky sexual behaviors among females.

## 3 Why Ghana

Access to reliable energy and energy services remains a challenge in developing nations, with a disproportionate impact on sub-Saharan African countries. Ghana has implemented programs to reduce dependence on traditional biomass, such as the National Liquefied Petroleum Gas (LPG) promotion campaign, however, the predominant sources of household cooking energy include firewood, followed by charcoal, gas, crop residue/sawdust, kerosene, then electricity (Adusah-Poku et al., 2019). While scholarship on the effect of household energy poverty on social and individual outcomes such as mental health (Lin and Okyere, 2020), and general well-being and health (Churchill & Smyth, 2021) are growing, the effect of energy poverty on important individual behaviors of public health significance, such as sex behavior has hardly been investigated.

Manu et al. (2022) reported that 79% and 68% of young women and men had unprotected sex during their last sexual intercourse. Although risky sexual behavior has been shown to be associated with poverty (specifically wealth) in Ghana, the link is far from consistent (Awusabo-Asare and Annim, 2008; Darteh, Dickson, & Amu, 2020). Given the youthfulness of Ghana's population, inquiries into how and the mechanism through which different forms of deprivation influence sexual behavior, particularly among individuals within the reproductive age bracket, are critical.

## 4 Methodology

### 4.1 Data

Consistent with our objective, we employed the 2014 Ghana Demographic and Health Survey (GDHS) by the Ghana Statistical Service. This nationally representative survey gathers information on respondents' energy services, health, and other socioeconomic characteristics via the DHS Phase VI core questionnaire. This survey employed a two-stage stratified sampling technique based on Ghana's 2010 Population and Housing Census data in sampling enumeration areas (EAs). Based on a probability sampling technique, 427 EAs were selected in the first stage of sampling, while a systematic sampling method was employed in determining households within the EAs in the second sampling stage. The Ghana Health Service Ethical Review Committee and the Institutional Review Board of ICF International, Maryland, USA, approved the 2014 GDHS. Interviewed households were those who provided informed consent before the interview. For our analysis, the household questionnaire provided vital information about household energy access and end usage, among other household factors, while the women's questionnaire covered specific questions on reproductive health and sexual behavior-related issues. General eligibility for the women's interview were women within the reproductive ages of 15–49 years. For this study, the analytical sample comprised unmarried female youth aged 15–34 years who had a history of sexual activity. The current study specifically focused on female youth as they are the ones noted to experience sexual violence and are more likely to engage in unprotected sex or have multiple sexual partners in exchange for resources as they lack control over sexual decision-making and are prone to contracting HIV (Dunkle et al., 2004; Kalichman & Simbayi, 2004; Weiser et al., 2006). Using this criterion, our analysis sample comprised 2445 observations.

### 4.2 Measurement of key variables

#### 4.2.1 Risky sexual behavior

Young women's risky sexual behavior is generally viewed as the occurrence and extent to which girls get involved in dangerous sexual activities (Christian et al., 2021). Following Christian et al. (2021), we captured this behavior based on four different indicators from the DHS. They involved 1) an individual engaging in early sex or involved in sexual activity before age sixteen; 2) an individual having unprotected sex (without a condom) with a non-marital or co-habiting partner within the last 12 months; 3) an individual having numerous partners she engaged with sexually in the past twelve months and 4) an individual being diagnosed of having contracted a sexually transmitted infection. Respondents who were not involved in either of the activities were coded 0 and referred to as “no risk”; those who engaged in at least one of these activities were coded 1 and referred to as “low risk”, while those involved in two or more were coded 2 and referred to as “high risk”.

#### 4.2.2 Multidimensional energy poverty

The Multidimensional Energy Poverty Index (MEPI) by Nussbaumer et al. (2012) was adopted in measuring energy poverty in this study. This is due to its ability to capture multiple energy deprivation and suitability in capturing energy poverty for developing economies (Lin & Okyere, 2020, Lin & Okyere, 2021; Nussbaumer et al., 2012). Following Nussbaumer et al. (2012), we assigned weights to the six indicators of the MEPI. These indicators included lighting, cooking, indoor air pollution, education/entertainment, household appliance ownership, and communication. Although equal weights can be assigned to these six dimensions, we apportioned relatively higher weights to the cooking, indoor air pollution, and lighting indicators due to their relative importance, especially for developing countries (Adusah-Poku & Takeuchi, 2019; Nussbaumer et al., 2013). Thus, weights of 0.205 were assigned to the cooking and indoor air pollution indicators, 0.200 to lighting, and an equal weight of 0.13 to the remaining three indicators. A household is considered deprived in an indicator: 1) if they are not connected to the grid for electricity; 2) if they use biomass fuels (firewood, charcoal, residue, etc.) for cooking or heating; 3) if they use the biomass fuel in an enclosed room that has no window or chimney; 4) if they do not own a refrigerator or deep freezer; 5) if they do not possess a TV or a radio set, and 6) if they do not own a mobile phone. We compute the MEPI by taking the weighted sum of these deprivations. The deprivation score involves ratios spanning from 0 to 1, where a score of 1 denotes households that are energy poor across all MEPI indicators, and 0 indicates the absence of energy poverty. This measure is denoted as EPOV1. Following the methodology outlined by Nussbaumer et al. (2012), we compare the deprivation score to a predefined threshold of 0.333. Households with scores equal to or exceeding this threshold are classified as multidimensionally energy poor, identified as EPOV2. Additionally, we implement MEPI cutoffs of 0.2 (EPOV3) and 0.5 (EPOV4), as suggested by Koomson and Danquah (2021) and Lin & Okyere, 2021. These measures, EPOV3 and EPOV4, contribute to the robustness of our estimates, aligning with methodologies established in prior studies (Lin & Okyere, 2022; Okyere et al., 2023; Okyere & Lin, 2023).

#### 4.2.3 Covariates

Following the related studies on risky sexual behavior, we controlled various variables that can influence the association between risky sexual behavior and energy poverty. Studies in sub-Saharan Africa have shown that low socioeconomic status is associated with inconsistent condom use, forced sex, and sex exchange (Dunkle et al., 2004; Gillespie & Kadiyala, 2005). We precisely controlled household income (Ali et al., 2021; Ali & Tadele, 2021; Awusabo-Asare & Annim, 2008), years of education (Ali et al., 2021; Christian et al., 2021; Gebeyehu & Mulatie, 2021), age (Ramjee & Daniels, 2013; Sieving et al., 2006), sex of household head (Gyan & Marhefka-Day, 2021; Watkins & Carson, 2021), and household size (Awusabo-Asare & Annim, 2008; Christian et al., 2021). We also accounted for location fixed effects by considering the locations within which these females were located. The definition and statistics of the variables used in this study are presented in Table 1.

**Table 1:** Description and summary statistics of the variables.

Variable	Description	Mean	SD
RSB	Risky sexual behavior (0 = no risk) (1 = low risk) (2 = high risk)	0.764	0.723
EPOV1	Energy deprivation score	0.570	0.288
EPOV2	Energy poverty based on 0.333 cutoff	0.780	0.413
EPOV3	Energy poverty based on 0.200 cutoff	0.764	0.723
EPOV4	Energy poverty based on 0.500 cutoff	0.764	0.723
Age	Age of female	25.383	5.087
Education	Years of education	7.079	4.478
Employed	Employment status (0 = unemployed) (1 = employed)	0.706	0.455
Dependent	Total number of children and elderly in the household	1.637	1.648
HH size	Household Size	4.811	2.733
Sex of HH head	Sex of household head (0 = male) (1 = female)	0.368	0.464
HH wealth	Household wealth terciles		
Poor	Poor (0 = non-poor, 1 = poor)	0.333	0.471
Middle Income	Middle Income household (0 = non-middle income, 1 = middle income)	0.333	0.471
Rich	Rich household (0 = non-rich, 1 = rich)	0.333	0.471
Location Fixed Effect	Location of residence (1 = Urban, 0 = Rural)	0.507	0.500

### 4.3 Estimation strategy

We estimated the relationship between energy poverty and risky sexual behavior following equation (1);

(1) $$RSB_{i}=\alpha_{o}+\beta_{1}EPOV_{i}+\sum_{n}\beta_{n}X_{n,i}+\delta_{i}+\varepsilon_{i}$$

Where RSB shows the female's risky sexual behavior; EPOV refers to energy poverty; $X_{n,i}$ is a vector of individual and household characteristics; $\varepsilon_{i}$ signifies the randomly distributed error term; $\beta_{n}$ represents the coefficients of the regressors; while the constant term and location fixed effect are defined by $\alpha_{o}$ and $\delta_{i}$, respectively.

We used the multinomial logistic regression model in analyzing the relationship between energy poverty and risky sexual behavior based on the fact that our dependent variable is categorical with three factors (i.e., “no risk”; “low risk,” and “high risk). The probability that a female youth i will engage in a risky sexual activity k is given by

(2) $$\mathrm{Pr}\left(y_{i}=k|x_{i}\right)=\{{\;}_{\frac{\exp\left(x_{i}\beta_{j}\right)}{1+\sum_{i=2}^{3}\exp\left(x_{i}\beta_{j}\right)}\;for\;k=2,3}^{\frac{1}{1+\sum_{i=2}^{3}\exp\left(x_{i}\beta_{j}\right)}\;for\;k=1}$$

where $x_{i}$ is a row vector of explanatory variables for female youth i, while $\beta_{k}$ represents the vector of coefficients for a risky sexual activity k. A detailed description of this technique can be found in Greene (2003). Due to the difficulty in interpreting the estimated coefficients using this model and the fact they do not represent changes in probabilities, marginal effects are usually computed as shown in equation (3);

(3) $$\frac{\partial \mathrm{Pr}\left(y_{i}=k|x_{i}\right)}{\partial x_{i}}=\mathrm{Pr}\left(y_{i}=k|x_{i}\right)\left[\beta_{k}-\sum_{i=2}^{3}\text{expPr}\left(y_{i}=j|x_{i}\right)\beta_{j}\right]$$

However, our estimates are likely to suffer from omission of relevant variables, reverse causality, or measurement errors. Hence, we employed the Lewbel 2-stage least regression to eliminate this endogeneity problem. Unlike GMM and IV regression, this technique presents an identification strategy founded on the heteroskedastic covariance restriction (Lewbel, 2012). Thus, this method does not rely on meeting the standard exclusion restrictions as it internally generates instruments for the endogenous variable (in our case, energy poverty). The technique has been widely employed (Churchill & Smyth, 2020; Koomson & Danquah, 2021; Zhang et al., 2021). In addition, we tested the robustness of our estimates by applying the propensity score matching method with various matching techniques and a sensitivity analysis with different cutoffs of energy poverty.

To further analyze the mediating role of total lifetime partners in explaining the impact of multidimensional energy poverty on risky sexual behavior, a two-step approach consistent with Koomson and Danquah (2021); Churchill and Smyth (2020) and Lin & Okyere, 2021 was applied. Following these studies, if the effect of energy poverty is reduced or rendered insignificant when total lifetime partners is introduced as a control variable, then total lifetime partners can be described as a mediating variable.

## 5 Results and discussion

### 5.1 Baseline results

Table 2 presents the estimates from the multinomial logistic regression model. Panel A and B present estimates where we regress risky sexual behavior on energy poverty captured using energy deprivation score (EPOV1) and the energy poverty cutoff (EPOV 2) based on Nussbaumer et al. (2012), respectively. Table 2 reveals that the coefficient of EPOV1 and EPOV2 in both panels is positive and significant statistically at the traditional significance level, indicating that energy deprivation is positively related to the probability of engaging in risky sexual behavior. Thus, for a percentage increase in energy deprivation, the probability of engaging in a lower and higher risky sexual activity increases by about 58% and 55%, respectively, holding other things constant (in Panel A). It can be noticed that this positive association remains consistent in Panel B. Thus, energy-poor individuals are about 35% and 36%, more likely to belong to low and high-risk categories of risky sexual activity, respectively, than their counterparts who do not involve themselves in these activities. This, shows that our baseline estimates are consistent with studies that have observed a positive association between risk activities and resource deprivation (e.g., Christian et al., 2021; McIlwaine & Moser, 2003; Noar et al., 2012). However, making causal inferences with our baseline results may be erroneous. We, therefore, present variant robust methods to resolve this issue, as presented in Table 3.

**Table 2:** Energy poverty and risky sexual behavior.

Variables	Panel A		Panel B	
	No risk Vs low risk	No risk Vs high risk	No risk Vs low risk	No risk Vs high risk
	(1)	(2)	(3)	(4)
EPOV1	0.580**	0.549*		
	(0.237)	(0.315)		
EPOV2			0.351***	0.355*
			(0.134)	(0.188)
Age	−0.103***	−0.170***	−0.108***	−0.170***
	(0.014)	(0.019)	(0.014)	(0.019)
Education (years)	0.007	−0.069***	0.007	−0.069***
	(0.013)	(0.019)	(0.013)	(0.019)
Employed	0.065	−0.110	0.064	−0.111
	(0.114)	(0.148)	(0.114)	(0.148)
Dependents	0.028	−0.157**	0.030	−0.155**
	(0.042)	(0.065)	(0.042)	(0.065)
HH size	0.063***	0.099***	0.061***	0.098***
	(0.020)	(0.025)	(0.020)	(0.025)
Sex of HH head	1.090***	1.638***	1.099***	1.646***
	(0.110)	(0.146)	(0.110)	(0.146)
HH wealth (Base: Poor)				
Middle Income	−0.082	−0.064	−0.158	−0.188
	(0.148)	(0.196)	(0.187)	(0.141)
Rich	−0.304	−0.469*	−0.405**	−0.560**
	(0.286)	(0.280)	(0.191)	(0.258)
Location Fixed Effect (base: Urban)	−0.121	−0.329*	−0.117	−0.325*
	(0.127)	(0.169)	(0.127)	(0.168)
Constant	2.283***	3.563***	2.447***	3.682***
	(0.465)	(0.620)	(0.420)	(0.565)

Observations	2445	2445	2445	2445

Standard errors in parentheses; ***p < 0.01, **p < 0.05, *p < 0.1.

**Table 3:** Lewbel IV estimates the effect of energy poverty on risky sexual behavior.

Variable	MEPI
***Panel A-Internal instrument***	
EPOV2	0.082*
	(0.045)
Covariates (?)	Yes
Cragg-Donald Wald F statistic	801.153
Observations	2353
***Panel*** B***-PSM***	
EPOV2	0.087**
	(0.050)
Covariates (?)	Yes
Observations	2353

Standard errors in parentheses; **p < 0.05, *p < 0.1.

### 5.2 Robustness checks

A series of tests are performed in this section to analyze the robustness of our results. As mentioned in Section 4.4, we present Lewbel IV and PSM estimates in Table 3. The estimates from the Lewbel IV reveal that energy deprivation increases a female's likelihood of engaging in risky sexual activity. Using internal instruments as presented in Panel A, female youth are about 8.2% highly likely to involve themselves in these risky sexual activities for a percentage increase in energy deprivation at a 10% significance level. Estimates from the propensity score matching using the regression adjustment matching technique as confirmatory analysis are also presented in Panel B of Table 3. The dependent variable's treatment effect on the treated (energy poor) is consistent with the baseline and Lewbel IV results. Specifically, female youth from energy-poor households are about 8.7% more likely to pursue risky sexual activities. Estimates from the Lewbel and PSM signify that the positive linkage between energy poverty and risky sexual behavior is consistent across the variant endogenous addressing techniques. It can also be observed that estimates from our baseline regression are greater in magnitude than the robust estimates, indicating that the effect of energy poverty on risky sexual activity is biased upward in the baseline regression.

Furthermore, we categorized our dependent variable into a dummy variable (engages or not) and estimated the effect of alternative cutoffs of energy poverty to check the sensitivity and robustness of our estimates, as presented in Table 4. In addition to the energy deprivation score (EPOV1) and energy poverty cutoff (EPOV 2) based on Nussbaumer et al. (2012), we apply a MEPI cutoff of 0.2 (EPOV3) and 0.5 (EPOV4) following Koomson and Danquah (2021), and Lin & Okyere, 2021. Except for EPOV 4, all other estimates from the alternative cutoffs of energy estimates confirm our initial findings, which suggest that energy poverty increases the involvement of female youths in these risky sexual acts.

**Table 4:** Sensitivity analysis.

Variable	Column A	Column B	Column C	Column D
EPOV1	0.114**			
	(0.046)			
EPOV2		0.072***		
		(0.025)		
EPOV3			0.073***	
			(0.027)	
EPOV4				0.034
				(0.025)
Covariates	Yes		Yes	Yes
Pseudo R2	0.124	0.125	0.124	0.122
Observation	2445	2445	2445	2445

Standard errors in parentheses; ***p < 0.01, **p < 0.05, *p < 0.1.

The positive effect of energy poverty and risky sexual acts supports hypothesis A and the broken window theory (Wilson & Kelling, 1982) that argues for normalcy in living in chaotic and disordered surroundings. An energy-poor household could mimic an environment comparable to a “broken window,” indicating neglect and deprivation. In such conditions, women are more likely to engage in risky sexual behavior due to the perceived lack of opportunities, resources, and social support. Additionally, the stress that may accompany energy poverty could lead to prioritizing immediate needs over long-term health considerations, influencing the decision-making process regarding sexual behavior. Specifically, females who are energy-poor are more likely to engage in these risky activities, as their environment (being energy-deprived) deprives them of vital information about the adverse effects of these activities. The lack of knowledge about contraceptives due to deprivation in end-usage appliances such as TV or radio contributes to their engagement in these risky activities. The deficiency in knowledge caused by being energy-deprived may lead to contraceptive failure – a significant cause of unintended pregnancies among youth in sub-Saharan Africa (Atiglo & Biney, 2021; Hindin et al., 2014). Additionally, a deprivation in energy solutions such as lighting could stall business and income generation, preventing women from becoming entrepreneurs and further increasing their dependence on sex for favors from men (Thorgren and Ghasemi Niavarani, 2021). Again, to escape the shame of being energy-deprived from not having mobile phones, females sometimes resort to these sexual activities to enable them to acquire energy services to maintain their social image (Day et al., 2016; Lin & Okyere, 2021; Middlemiss et al., 2019).

### 5.3 Heterogeneity analysis

Subsequently, we probe the impact of energy poverty on risky sexual activity across variant sub-samples. The idea of performing the analysis at variant levels of the data is consistent with the principal goal of the SDGs of “leaving no one behind.” Table 5 presents the energy poverty-risky sexual behavior nexus across income and location subgroups. Our estimates reveal that the association between energy poverty and risky sexual behavior is positive across the income and location subgroups but not significant consistently across all the subgroups. The results show that EPOV1 and EPOV2 positively and significantly affect the risky sexual behavior of female youths from poor homes and rural areas. For instance, energy-poor females from poor homes are about 27% more likely to involve themselves in these risky activities than their non-energy-poor counterparts at a 5% significance level. The outcome of a higher likelihood of energy-poor girls belonging to the poorer income terciles involving themselves in risky sexual activities is consistent with other studies (Awusabo-Asare & Annim, 2008; Christian et al., 2021). This is because females from wealthier homes have access to TV or radio that enables them to access relevant information about the benefits of safe sex. Hence, they will opt for condoms during sexual intercourse, unlike their energy-poor counterparts.

**Table 5:** Heterogenous analysis.

Variable	Panel A: Income Tercile			Panel B: Location	
	Poor	Middle	Rich	Rural	Urban
EPOV1	0.183*	0.089	0.035	0.178*	0.028
	(0.109)	(0.121)	(0.133)	(0.093)	(0.100)
EPOV2	0.271**	0.116	0.051	0.225***	0.004
	(0.127)	(0.085)	(0.049)	(0.069)	(0.045)
Covariates	Yes	Yes	Yes	Yes	Yes

Standard errors in parentheses; ***p < 0.01, **p < 0.05, *p < 0.1.

Similarly, energy-poor females from rural areas are about 22.5% more likely to participate in risky sexual activities than their non-energy-poor folks, at a 1% significance level. This is due to the deprivations in end-use appliances such as television or radio (Lin & Okyere, 2020; Zhao et al., 2010) that would have enabled these rural energy-poor females to obtain credible information on protective measures such as seeking treatments for sexual infections or using a condom (Lopman et al., 2007). In a study across 14 sub-Saharan African countries, the number of rural adolescents who reported engaging in sex without a condom was about 20% higher than their counterparts in urban areas (Ali et al., 2021). With energy poverty being prevalent in these rural settings (Adusah-Poku & Takeuchi, 2019; Lin & Okyere, 2020), the lack of information via end-use appliances increases the chances of energy-poor females in rural areas engaging in these risky acts.

### 5.4 Mechanism

Mediums via which energy poverty affects risky sexual behaviors are presented in Table 6. Based on the nature of the dataset, we concentrate on one mechanism: the total number of lifetime partners. Panel B of Table 6 shows that an increase in energy poverty positively and significantly impacts the total number of partners the respondents have had in their lifetime. After the positive association between energy poverty and risky behaviors among female youth, the impacts of the total number of life partners are included in the model displayed in Panel C of Table 6. A decline in the magnitude of energy poverty compared to that of Panel A can be observed when the mediating variable is included in Panel C. Therefore, we can conclude and confirm that the total number of lifetime partners plays a significant role in aggravating the impact of energy poverty on females’ engagement in risky sexual activities.

**Table 6:** Mediation analysis.

	Dependent Variable		
	Panel A	Panel B	Panel C
	Risky Sexual Behavior	Life time partners	Risky Sexual Behavior
EPOV2	0.111***	0.021*	0.083**
	(0.042)	(0.012)	(0.037)
Lifetime partners			0.019***
			(0.007)
Covariates (?)	Yes	Yes	Yes
R-squared	0.237	0.024	0.224
Observation	2445	1040	2445

Standard errors in parentheses; ***p < 0.01, **p < 0.05, *p < 0.1.

The mediation analysis is consistent with hypothesis B and the literature that views risky sexual activities as a coping strategy (Gillespie & Kadiyala, 2003, 2005; Weiser et al., 2007). These theories view people's involvement in these activities as eradication schemes for resource deprivation. Thus, when women are deprived of energy services, they are likely to resort to multiple older men for material or economic advantage (Mill & Anarfi, 2002; Weiser et al., 2007). In exchange for the energy resource, these men demand sex from the females. In addition, with the mental stressors that come with energy poverty, some females veer into having numerous partners as a maladaptive coping strategy and end up involving themselves in riskier sex (Briere, 2004). This is because their involvements in riskier sex may be relatively cheap and guarantee welfare gains as the benefits from energy services provided by the numerous partners augment the risk of having unprotected sex.

## 6 Conclusion and policy implications

The socioeconomic costs brought unto society at all levels resulting from energy poverty and risky sexual activities are substantial. Risky sexual behavior has been argued to be another significant socioeconomic problem given its consequences for unintended pregnancies and sexually transmitted diseases among the youth. However, the interplay between these issues is yet to be explored. This paper tests the influence of energy deprivation on female youth involvement in risky sexual activities using the 2014 Demographic and Health Survey in Ghana. Interestingly, the estimates show that energy deprivation increases the likeliness of female youths engaging in risky sexual activities. This result is consistent across poorer and rural subgroups and alternative cutoffs of energy poverty. We further identified channels through which energy poverty influences risky sexual activity. Specifically, we find the total number of lifetime partners as a virtual channel through which energy poverty influences risky sexual activity among female youth.

The results elicit essential policy recommendations for advocates and decision-makers. These outcomes call for sexual health policies to recognize the role of energy deprivation play among the targeted populations. Although the Ghana Adolescent Health Service Policy and Strategy 2016–2020 (GHS, 2016) has identified sexual and reproductive health as fundamental problems that require attention, these policies usually lack recommendations that consider the adverse effects of energy deprivation. Therefore, the implementation of existing reproductive and sexual health programs ought to consider the association between energy deprivation and sexual behavior.

Furthermore, strategies promoting affordable energy services to poor and rural households could effectively help address the adverse effects of energy poverty on sexual behavior. An expanded lighting initiative in rural areas via the provision of solar street lighting and lanterns can help break the normalcy of risky sexual activities that come with living in energy-deprived environments. Rural-based enterprises can be set up to sell rechargeable solar lights and charge mobile phones using the solar photovoltaic (PV) systems provided at a rate above the marginal cost to earn additional revenue. This limits female youths from resorting to adult men for their needs. This scheme also serves as a service rather than an upfront purchase, enabling low-income families to address energy affordability issues. End-usage appliances can also be provided to girls from energy-deprived households to allow them to access educative programs on the adverse effect of risky sexual activities to limit their involvement. Moreover, the analysis on the channel of influence suggests that counseling, especially for female youth on multiple partners, could dampen the effect of energy poverty on sexual behavior among youth.

Despite the robustness of the study's estimates, the study employed cross-sectional data for the analysis due to the unavailability of panel data. This makes it impossible to discuss unobserved characteristics of the female youth. Additionally, we acknowledge the complex nature and drivers of risky sexual behavior and thus suggest further studies, including qualitative and mixed-method studies, that could provide more context to the energy poverty and sexual behavior discourse. This notwithstanding, this study provides exciting insight, paving the way for further research on the topic. Future research may consider a panel analysis and explore more channels of influence.

## Ethical approval

The Ghana Health Service Ethical Review Committee and the Institutional Review Board of ICF International, Maryland, USA, approved the 2014 GDHS. Interviewed households were those who provided informed consent before the interview.

## Funding

There was no funding associated with this work.

## Ethical statement

The Ghana Health Service Ethical Review Committee and the Institutional Review Board of ICF International, Maryland, USA, approved the 2014 GDHS. Interviewed households were those who provided informed consent before the interview. The study questionnaire was explained clearly to all eligible participants at the time of recruitment, and each participant signed or, if illiterate, thumb-printed an informed consent document before being enrolled in the study.

## CRediT authorship contribution statement

**Michael Adu Okyere:** Writing – original draft, Methodology, Formal analysis, Conceptualization. **Aaron Kobina Christian:** Writing – original draft, Methodology, Conceptualization. **Felix Essel-Gaisey:** Writing – original draft, Methodology, Formal analysis, Data curation, Conceptualization. **Fawzia Muhammed Zuka:** Writing – original draft, Methodology, Formal analysis, Data curation.

## Declaration of competing interest

The authors declare that they have no known competing financial interests or personal relationships that could have influenced the work reported in this paper.

## Data Availability

Data will be made available on request.
